# Arterial function, biomarkers, carcinoid syndrome and carcinoid heart disease in patients with small intestinal neuroendocrine tumours

**DOI:** 10.1007/s12020-022-03065-0

**Published:** 2022-05-10

**Authors:** Iiro Kostiainen, Noora Karppinen, Piia Simonen, Milla Rosengård-Bärlund, Riikka Lindén, Maija Tarkkanen, Daniel Gordin, Janne Rapola, Camilla Schalin-Jäntti, Niina Matikainen

**Affiliations:** 1 Endocrinology, Abdominal Center, Helsinki University Hospital and University of Helsinki, Helsinki, Finland; 2 Cardiology, Heart and Lung Center, Helsinki University Hospital and University of Helsinki, Helsinki, Finland; 3 Radiology, HUS Diagnostic Center, Helsinki University Hospital and University of Helsinki, Helsinki, Finland; 4 Comprehensive Cancer Center, Helsinki University Hospital and University of Helsinki, Helsinki, Finland; 5 Finnish Medicines Agency FIMEA, Helsinki, Finland; 6 Department of Nephrology, Abdominal Center, Helsinki University Hospital and University of Helsinki, Helsinki, Finland; 7 Minerva Institute for Medical Research, Helsinki, Finland

**Keywords:** Arterial function, Carcinoid heart disease, Carcinoid syndrome, Small intestinal neuroendocrine tumour, Transthoracic echocardiography

## Abstract

**Purpose:**

Carcinoid heart disease (CHD) is a life-threatening complication of carcinoid syndrome (CS) characterised by tricuspid regurgitation (TR). However, there is an unmet need for earlier diagnosis of CHD. We cross-sectionally assessed the prevalence and potential predictive or diagnostic markers for CS and CHD in a contemporary cohort of patients with small intestinal neuroendocrine tumours (SI-NETs).

**Methods:**

Biochemical characteristics, hepatic tumour load, measures of arterial and endothelial function, atherosclerosis, and transthoracic echocardiography were analysed in a prospective cross-sectional setting.

**Results:**

Among the 65 patients studied, 29 (45%) had CS (CS+ ), and 3 (5%) CHD. CS+ was characterised by significantly higher hepatic tumour load, S-5-HIAA and fP-CgA, higher frequency of diarrhoea and flushing, and more frequent PRRT compared to CS− (for all, *P* < 0.05). Central systolic, central mean, and central end-systolic blood pressures were significantly higher in CS+ than in CS− (for all, *P* < 0.05). Subjects with grades 2–4 TR had higher hepatic tumour burden, fP-CgA, and S-5-HIAA compared to those with grades 0–1 TR, but measures of vascular function did not differ. fP-CgA (*P* = 0.017) and S-5-HIAA (*P* = 0.019) but not proBNP increased significantly according to the severity of TR.

**Conclusion:**

Although CS is common, the prevalence of CHD was found to be lower in a contemporary cohort of SI-NET patients than previously anticipated. Measures of arterial or endothelial function or carotid atherosclerosis do not identify subjects with mild TR. Echocardiography remains the most sensitive means to diagnose CHD in CS patients with high tumour burden and elevated CgA and 5-HIAA.

## Introduction

The incidence of small intestinal neuroendocrine tumours (SI-NETs) originating from the enterochromaffin cells in the ileum (midgut) has risen in recent years [1]. In the 2012 SEER database, the incidence was 1.3/100 000 [2]. Advanced disease that is usually metastatic often leads to carcinoid syndrome (CS). CS lacks a standard definition but is characterised by typical symptoms of diarrhoea, flushing, bronchial constriction and elevated concentrations of serotonin and serotonin metabolites, the most important of which is 5-hydroxyindoleacetic acid (5-HIAA) [3, 4]. SI-NETs with substantial tumour burden and high levels of circulating serotonin and its metabolites are associated with carcinoid heart disease (CHD), a condition that has been found to cause substantial morbidity and mortality in the form of right-sided heart failure [1]. In contemporary patient series, frequency of CHD among patients with CS varies between 21 and 37% [5, 6].

The pathophysiology of valvular injury in CHD is yet to be fully understood [1]. Previous studies have identified high circulating serotonin concentrations as the major culprit behind the development of CHD. This relationship has been revealed in animal studies, in which the long-term administration of high-dose serotonin or the deficiency of the 5-HIAA transporter gene has been shown to result in the formation of carcinoid-like plaques on cardiac valves [7, 8]. Furthermore, a stimulatory action of serotonin on subendocardial cell proliferation has been demonstrated in cell culture studies [9]. In humans, the essential role of serotonin in the development of CHD is based on indirect evidence only. Human heart valves express mRNA for serotonin receptors (5-HT1B, 1D, 2 A, and 2B) [10]. In clinical studies, concentrations of urinary 5-HIAA have been found to be higher among patients with CHD than those without cardiac involvement. Furthermore, increased urinary 5-HIAA levels have been associated with the progression of CHD [11].

Aside from the cardiac valves, endothelial cells also express various serotonin receptors [12, 13]. These serotonin receptors are important controllers of arterial function and tone and mediate both vasoconstrictive and vasodilatory arterial effects under normal metabolic conditions [14–16]. Therefore, a long-term excess of circulating serotonin could affect vascular endothelial function. In addition to serotonin, SI-NETs can also produce other vasoactive substances, such as substance P, neurokinin A, neuropeptide K, histamine, prostaglandins, bradykinin, activin A, connective tissue growth factor, and transforming growth factor beta [1]. These, or other yet unrecognised humoral agents, may be alternative aetiological factors or may act synergistically with serotonin to induce the lesions found in the heart and possibly elsewhere in the circulatory system. However, it remains unknown whether patients with SI-NETs are characterised by impaired endothelial function.

Although early diagnosis and timely surgical intervention in CHD patients offer a survival benefit, the diagnosis is based on echocardiography findings of irreversible fibrosis related right-sided valvular deformation with frequent tricuspid regurgitation and pulmonary regurgitation/stenosis [17–19]. More pronounced valvular dysfunction may lead to increased right ventricular strain and eventual right-sided heart failure. CHD is associated with diminished survival [20], with a recent study demonstrating an approximately two-year reduction in median survival when compared to patients characterized by CS only [5].

There is thus a need for earlier diagnosis via specific prognostic markers or novel diagnostic tools for CHD, which is currently detected in a late phase mostly after irreversible right-side valvular damage has occurred. The aim of our study was to evaluate the prevalence of CHD and study possible predictive biochemical and cardiovascular markers for CS and CHD in a contemporary cohort of patients with SI-NET at a tertiary centre. Our aim was also to find possible subtle alterations in biomarkers, arterial function measurements, or echocardiography that could represent early diagnostic markers for CHD.

## Patients and methods

### Patients and study design

The study population included patients with a histologically confirmed diagnosis of SI-NET who were treated at the Helsinki University Hospital in the Departments of Endocrinology and Oncology between May 2016 and November 2017. All participants gave their written informed consent. Subjects with hereditary tumour predisposition syndromes were excluded. The study was conducted in accordance with the Declaration of Helsinki and approved by the Ethics Committee of Helsinki University Hospital.

This was a prospective, cross-sectional study including arterial function measurements, transthoracic echocardiography, and biochemical measurements of chromogranin A (fP-CgA), urinary and serum 5-HIAA and P-proBNP of the patients. The most recent imaging study performed as part of the clinical follow-up was used to assess hepatic tumour burden. A questionnaire assessing CS symptoms was performed at the study visit. Prevalence of tumour-related symptoms, duration of disease, treatment modalities, and pathology reports were retrieved from the electronic patient records.

CS was defined as the presence of related symptoms (i.e., diarrhoea, flushing, or CHD) in conjunction with S-5-HIAA concentrations above the upper limit of normal (ULN, reference range <123 nmol/l) or S-5-HIAA concentrations higher than three times the ULN, regardless of symptoms.

### Laboratory methods

All laboratory analyses were performed at HUSLAB, the laboratory of Helsinki University Hospital. FP-CgA was measured with immunoradiometric assay, urinary and serum 5-HIAA with liquid chromatography-mass spectrometry [21], and P-proBNP with immunochemiluminometric assay. S-5-HIAA and fP-CgA were available from all patients. Urinary 5-HIAA was available from 36 patients (55%) and proBNP from 63 patients (97%) because of logistic problems. Primary tumours were graded according to the 2019 WHO classification of tumours of the digestive system using pathology report data [22].

### Measures of arterial function

#### Arterial stiffness

Arterial stiffness was measured by applanation tonometry from the radial artery with a pen-like micromanometer (SPC-301; Millar Instruments, Texas, USA). A model of the central pressure waveform was synthesised with SphygmoCor software (SphygmoCor; ATCOR Medical, Sydney, Australia) using a validated generalised transfer function as previously described [23]. A mean of two measurements was used in the analysis.

Pulse wave velocity (PWV) pressure waveforms were recorded sequentially at the carotid, femoral, and radial arteries to measure arterial stiffness in the large (aortic) and intermediate (brachial)-sized arteries. With a simultaneous ECG recording of the R wave as a reference frame, the system software calculated the PWV [24]. The differences in the carotid to femoral and carotid to radial path length were estimated from the distance from the sternal notch to the femoral and carotid palpable pulse.

The augmentation index (AIx) and subendocardial viability ratio (SEVR) were derived from measurements. AIx, adjusted at 75 beats per minute, is the most commonly used parameter for arterial stiffness in small arteries (resistance vessels). SEVR was used to approximate subendocardial perfusion of the heart.

#### Carotid intima-media thickness

Intima-media thickness (IMT) of the common carotid arteries (CCA) was measured with a multiarray echo tracking system (ArtLab) based on classical high-resolution echo tracking technology (WallTrack system), which generates high precision and reproducibility [25, 26]. Measurements were performed on both the right and left CCA. A mean of two measurements was used in the analysis.

#### Endothelial function

The reactive hyperaemia index (RHI), a measure of endothelial function, was calculated using measurements from a peripheral arterial tonometry (PAT) device placed on the tip of each index finger (Endo-PAT2000, Itamar Medical, Caesarea, Israel). The PAT device applies uniform pressure to the surface of the distal finger, allowing for measurement of pulse volume changes in the finger [27]. Baseline pulse amplitude was measured from each fingertip for 5 minutes. Arterial flow was interrupted for 5 minutes by a cuff placed on a proximal forearm (Hokanson AG101, D.E. Hokanson Inc., Bellevue, WA, USA) at whichever occlusion pressure was higher between 200 mm Hg and 60 mm Hg plus systolic blood pressure. Pulse amplitude was recorded electronically in both fingers and analysed by a computerised, automated algorithm (Itamar Medical) that provided the average pulse amplitude for each 30-second interval after forearm cuff deflation for up to 5 minutes. To evaluate the vascular response in relation to baseline, with adjustment for systemic effects and skewed data, the hyperaemic response was expressed as the natural logarithm of the ratio of post-deflation to baseline pulse amplitude in the hyperaemic finger divided by the same ratio in the contralateral finger, which served as a control.

### Echocardiography

Transthoracic echocardiography (TTE) was performed on the high-end Philips EPIQ 7 cardiac ultrasound system (Philips Ultrasound Inc., Bothell, WA, USA) using the X5-1 Matrix Array Transducer. Recordings were saved in DICOM format for analysis with QLab (Philips Medical Systems).

TTE examinations were carried out in a standard manner with the patient in the supine left lateral position. Standard parasternal long-and short-axis views, together with apical 4-, 3-, and 2-chamber views, were recorded. An additional parasternal long axis view for optimal visualisation of the tricuspid valve was recorded. Pulsed wave Doppler was used to measure the tricuspid inflow. Continuous wave Doppler was used for measuring the tricuspid regurgitation (TR) gradient as well as the pulmonary valve gradient. Colour Doppler was used for evaluating tricuspid and pulmonary valve regurgitation. Two cardiologists evaluated the echocardiographic imaging results afterwards without knowing the results of each other’s analysis.

The diagnosis of CHD was based on echocardiographic grading of TR, leaflet mobility, and morphological abnormalities of the leaflets. From these parameters, we calculated a score that has shown good discrimination between individuals with and without CHD [28]. A score of three was used as a cut-off point for CHD, with previous work showing high sensitivity and specificity [29].

### Assessment of hepatic tumour burden

An abdominal radiologist (R.L.) reassessed the hepatic tumour burden of the SI-NET patients using available radiologic follow-up imaging, which was tailored to the individual treatment scheme. Assessment was based on CT (*n* = 47, 72.3%), MRI (*n* = 13, 20.0%), or ^68^Ga-DOTANOC-PET-CT (*n* = 5, 7.7%). Tumour burden was estimated using a visual semi-quantitative approach. This method has previously been applied in other studies on patients with NETs [30, 31]. Four to six scan slices with the most extensive disease burden were selected and scored visually. Hepatic tumour burden was divided into five categories: 0, <10, 10–25, 25–50, and >50%.

### Statistical analysis

Data are presented as means and standard deviation for continuous variables and medians and ranges for non-normally distributed variables. Proportions were calculated for categorical data. Statistical analysis was performed with IBM SPSS Statistics 25. The chi-squared test was used to calculate differences in the categorical variables between groups. Mean ranks between groups were compared using the Mann–Whitney U test or the Kruskal–Wallis test for comparison of more than two groups. Correlations were analysed with Pearson’s correlation coefficient. Stepwise logistic regression was performed with binary logistic regression with a forward conditional method. Reported *P* values are two-sided, with a *P* value of <0.05 considered statistically significant.

## Results

### Basic characteristics

Of the 65 patients with SI-NET, 55% (46/65) did not have CS (CS−), 45% had CS (CS+ ), and 5% (3/65) had CHD. The mean age, disease duration, BMI, and gender distribution did not differ between the groups. The patient characteristics are described in Table 1. Fifty-eight (89%) of the patients had at least locally spread disease, 51 (78%) had distant metastases, and 50 (77%) had liver metastases. The frequency of both distant metastases in general and liver metastases was significantly higher in CS+ patients as compared to those who were CS− (Table 1).

**Table 1:** Patient characteristics

Variable	All patients (*n* = 65)	CS− (*n* = 36)	CS+ (n = 29)	*p* value (comparison to CS−)	CS+ excluding CHD (*n* = 26)	*p* value (comparison to CS−)	CHD (*n* = 3)
Age (years)	64.2 ± 8.9	64.7 ± 8.8	63.6 ± 9.1	0.649	64 ± 9.4	0.836	59.7 ± 5.5
Sex (male/female)	33/32 (51/49%)	19/17 (53/47%)	14/15 (48/52%)	0.805	12/14 (46/54%)	0.797	2/1 (67/33%)
BMI	26.6 ± 4.7	27.2 ± 4	25.9 ± 5.5	0.141	26.4 ± 5.5	0.343	21.4 ± 2.9
Duration of disease (months)	76 ± 58	83 ± 62	67 ± 53	0.300	72 ± 53	0.583	20 ± 14
Primary tumour Ki-67 (%)	3.2 ± 3.1	3.3 ± 2.9	3.1 ± 3.3	0.588	3.1 ± 3.4	0.578	3 ± 2.8
Primary tumour grade^a^							
NET G1	41 (67%)	21 (64%)	20 (71%)		19 (73%)		1 (50%)
NET G2	20 (33%)	12 (36%)	8 (29%)		7 (27%)		1 (50%)
NET G3	0 (0%)	0 (0%)	0 (0%)		0 (0%)		0 (0%)
CgA (nmol/l)	5 (1.6–4100)	2.9 (1.6–16)	19 (4.2–4100)	**<0.001**	16 (4.2–4100)	**<0.001**	250 (96–400)
Serum 5-HIAA (nmol/l)	138 (37–7470)	79 (37–582)	442 (132–7470)	**<0.001**	405 (132–7170)	**<0.001**	3220 (1940–7470)
Urinary 5-HIAA (µmol/24 h)	32 (11–1621)	25 (11–127)	84 (19–1621)	**0.001**	76 (19–1621)	**0.001**	1354 (1354)
proBNP (ng/l)	81 (7–5907)	55 (16–1573)	93 (7–5907)	0.146	87 (7–1175)	0.296	1283 (86–5907)
Locally advanced or metastatic disease	58 (89%)	29 (81%)	29 (100%)	**0.014**	26 (100%)	**0.035**	3 (100%)
Any distant metastases	51 (78%)	24 (67%)	27 (93%)	**0.014**	24 (92%)	**0.029**	3 (100%)
Liver metastases	50 (77%)	24 (67%)	26 (90%)	**0.040**	23 (89%)	0.122	3 (100%)
Hepatic tumour load				**<0.001**		**0.001**	
0%	23 (35.4%)	19 (52.8%)	4 (13.8%)		4 (15.4%)		0 (0%)
1–10%	23 (35.4%)	12 (33.3%)	11 (37.9%)		11 (42.3%)		0 (0%)
11–25%	9 (13.8%)	4 (11.1%)	5 (17.2%)		4 (15.4%)		1 (33.3%)
26–50%	7 (10.8%)	1 (2.8%)	6 (20.7%)		6 (23.1%)		0 (0%)
>50%	3 (4.6%)	0 (0%)	3 (10.3%)		1 (3.8%)		2 (66.7%)
Flushing	23 (35%)	8 (22%)	15 (52%)	**0.019**	13 (50%)	**0.031**	2 (67%)
Diarrhoea	39 (60%)	17 (47%)	22 (76%)	**0.024**	20 (77%)	**0.035**	2 (67%)
Bowel movements/day	3.8 (1.0–7.0)	1.0 (0.8–5.3)	5.5 (3.0–7.0)		5.3 (2.6–7.0)		7.0 (7.0–7.0)
Somatostatin analogue treatment	58 (89%)	30 (83%)	28 (97%)	0.120	25 (96%)	0.222	3 (100%)
Peptide receptor radionuclide therapy	24 (37%)	8 (22%)	16 (55%)	**0.010**	15 (58%)	**0.007**	1 (33%)
Interferon therapy	12 (18%)	4 (11%)	8 (28%)	0.114	8 (31%)	0.101	0 (0%)
Primary tumour resected	58 (89%)	33 (92%)	25 (86%)	0.691	25 (96%)	0.633	0 (0%)
Liver metastases resected	12 (19%)	8 (22%)	4 (14%)	0.524	4 (15%)	0.538	0 (0%)
Other metastases resected	14 (22%)	8 (22%)	6 (21%)	1.000	6 (23%)	1.000	0 (0%)
Liver thermoablation	3 (5%)	3 (8%)	0 (0%)	0.247	0 (0%)	0.258	0 (0%)

Values are presented as means ± SD, median (range), or *n* (proportion), as appropriate. Emphasis in bold denotes statistical significance (*P* < 0.05)

^a^Graded according to the 2019 WHO classification of tumours of the digestive system [22]. Primary tumours for grading were available from 61 subjects

### Treatments for SI-NET

The primary tumour had been resected in 58 (89%) of the patients. Twelve (19%) patients had undergone surgical resection of hepatic metastases. Resection of other metastases (including lymph node metastases) was performed in 14 (22%) patients. Three patients (5%) had received liver thermoablation therapy.

Fifty-eight patients (89%) were on somatostatin analogue treatment, 24 (37%) had undergone peptide receptor radionuclide therapy (PRRT; with ^177^Lu-DOTATATE), with a median of 6.5 cumulative cycles (range 3–10), 12 (18%) received interferon alpha, and three (5%) mTOR inhibitor or chemotherapy (everolimus, temozolomide, or temozolomide in combination with capecitabine). CS+ patients had received PRRT more often than those who were CS− (15/26 (58%) vs. 8/36 (22%), *P* = 0.01).

### Hepatic tumour burden, symptoms, and biomarkers in patients with and without CS

CS+ patients had a significantly higher hepatic tumour burden (*P* < 0.001, distribution is shown in Table 1) when compared to those who were CS−. Prevalence of flushing (50% vs. 8%, *P* = 0.031) and diarrhoea (77% vs. 47%, *P* = 0.035) and concentrations of S-5-HIAA (838 ± 1459 nmol/l vs. 101 ± 89 nmol/l, P < 0.001), dU-5-HIAA (226 ± 451 µmol vs. 32.4 ± 24.8 µmol, *P* = 0.001), and fP-CgA (184 ± 800 nmol/l vs. 3.8 ± 2.7 nmol/l, *P* < 0.001) were higher in CS+ patients as compared to those who were CS−. The inclusion of the three CHD patients in the analysis did not change the results (Table 1).

When all patients were included in the analysis, S-5-HIAA correlated significantly with tumour load (r = 0.582, *P* < 0.001), fP-CgA (r = 0.677, P < 0.001), P-proBNP (r = 0.597, *P* < 0.001) and AIx (r = −0,264, *P* = 0.035). When CHD patients were excluded from the analysis, S-5-HIAA correlated significantly only with tumour load (r = 0.454, *P* < 0.001), and fP-CgA (r = 0.883, *P* < 0.001).

### Measures of arterial function

The results of the arterial function measurements are described in Table 2. When CHD patients were excluded, CS+ patients had significantly higher central systolic pressure (136 ± 18 mmHg vs. 127 ± 15 mmHg, *P* = 0.042), central mean pressure (105 ± 11 mmHg vs. 99 ± 11 mmHg, *P* = 0.029), and central end-systolic pressure (121 ± 15 mmHg vs. 113 ± 13 mmHg, *P* = 0.030) compared to those who were CS−. Of note, the carotid IMT and endothelial function (RHI) did not differ between the groups.

**Table 2:** Arterial function measurements

Variable	All patients (*n* = 65)	CS− (*n* = 36)	CS+ (*n* = 29)	CS+ excluding CHD (*n* = 26)	CHD (*n* = 3)
Subendocardial viability ratio, SEVR	151.8 ± 25.6	151.9 ± 29	151.6 ± 21.1	149.4 ± 20.7	170.6 ± 16.5
Augmentation index, C-APHG HR75 AIx	23.9 ± 9.2	23.7 ± 8.8	24.1 ± 9.7	25.4 ± 8.9	13.1 ± 11.4
Central systolic pressure (mmHg)	130 ± 17	127 ± 15	133 ± 20	**136** ± **18**	109 ± 20
Central diastolic pressure (mmHg)	80 ± 10	78 ± 9	81 ± 11	83 ± 10	67 ± 12
Central mean pressure (mmHg)	100 ± 12	99 ± 11	103 ± 13	**105** ± **11**	83 ± 12
Brachial pulse wave velocity (m/s)	8.5 ± 1.2	8.3 ± 1.1	8.6 ± 1.3	8.6 ± 1.3	8.9 ± 0.9
Aortic pulse wave velocity (m/s)	9.7 ± 2.7	9.8 ± 3	9.7 ± 2.5	9.8 ± 2.6	8.6 ± 1.9
Reactive hyperaemia index, RHI	2.2 ± 0.7	2.3 ± 0.7	2.1 ± 0.7	2.2 ± 0.8	1.7 ± 0.3
Intima-media thickness, left mean (µm)	683 ± 133	693 ± 140	670 ± 124	679 ± 129	601 ± 11
Intima-media thickness, right mean (µm)	633 ± 134	633 ± 149	634 ± 114	649 ± 108	500 ± 73
Mean common carotid artery diameter (mm)	7.7 ± 1	7.7 ± 1.1	7.6 ± 0.9	7.7 ± 0.9	6.8 ± 0.3

Values are presented as mean ± SD. Emphasis in bold denotes statistical significance (*P* < 0.05) when compared to CS− group

### Echocardiography

Three patients were diagnosed with CHD.

Selected TTE results related to the function and anatomy of the right side of the heart are presented in Table 3. Additional variables evaluated included thickening, mobility, and regurgitation of the aortic and mitral valves and stenosis of the aortic, mitral, and pulmonic valves.

**Table 3:** Transthoracic echocardiographic measurements related to right side of the heart

Variable	All patients	CS− (*n* = 36^b^)	CS+ (*n* = 29^b^)	CS+ excluding CHD (*n* = 26^b^)	CHD (*n* = 3)
Tricuspid valve regurgitation					
None	9 (14.8%)	8 (23.5%)	1 (3.7%)	1 (4.2%)	0 (0%)
Trace	23 (37.7%)	13 (38.2%)	10 (37%)	10 (41.7%)	0 (0%)
Mild	24 (39.3%)	12 (35.3%)	12 (44.4%)	12 (50%)	0 (0%)
Moderate	3 (4.9%)	1 (2.9%)	2 (7.4%)	1 (4.2%)	1 (33.3%)
Severe	2 (3.3%)	0 (0%)	2 (7.4%)	0 (0%)	2 (66.7%)
Pulmonic valve regurgitation					
None	39 (63.9%)	24 (72.7%)	15 (53.6%)	15 (60%)	0 (0%)
Trace	6 (9.8%)	1 (3%)	5 (17.9%)	5 (20%)	0 (0%)
Mild	13 (21.3%)	8 (24.2%)	5 (17.9%)	5 (20%)	0 (0%)
Moderate	1 (1.6%)	0 (0%)	1 (3.6%)	0 (0%)	1 (33.3%)
Severe	2 (3.3%)	0 (0%)	2 (7.1%)	0 (0%)	2 (66.7%)
Tricuspid valve leaflet mobility					
Increased	0 (0%)	0 (0%)	0 (0%)	0 (0%)	0 (0%)
Normal	58 (93.5%)	35 (100%)	23 (85.2%)	23 (95.8%)	0 (0%)
Mildly reduced	2 (3.2%)	0 (0%)	2 (7.4%)	1 (4.2%)	1 (33.3%)
Moderately reduced	1 (1.6%)	0 (0%)	1 (3.7%)	0 (0%)	1 (33.3%)
Severely reduced	1 (1.6%)	0 (0%)	1 (3.7%)	0 (0%)	1 (33.3%)
Pulmonic valve leaflet mobility					
Increased	0 (0%)	0 (0%)	0 (0%)	0 (0%)	0 (0%)
Normal	56 (94.9%)	32 (100%)	24 (88.9%)	24 (100%)	0 (0%)
Mildly reduced	2 (3.4%)	0 (0%)	2 (7.4%)	0 (0%)	2 (66.7%)
Moderately reduced	0 (0%)	0 (0%)	0 (0%)	0 (0%)	0 (0%)
Severely reduced	1 (1.7%)	0 (0%)	1 (3.7%)	0 (0%)	1 (33.3%)
Right ventricle area, systolic (cm^2^)^a^	12 ± 4.5	11.8 ± 4.7	12.4 ± 4.4	12.5 ± 4.4	11.2 ± 5.3
Right ventricle basal dimension, diastolic (mm)^a^	35.1 ± 6	35.3 ± 6.4	34.8 ± 5.5	34.1 ± 5	40 ± 7.9
Right ventricle mid-cavity dimension, diastolic (mm)^a^	31.6 ± 6.4	32.2 ± 6.4	30.9 ± 6.4	30.7 ± 6.2	32 ± 9.8
Right ventricle longitudinal dimension, diastolic (mm)^a^	62.8 ± 7.1	62.6 ± 7.7	63 ± 6.5	62.9 ± 6	64.3 ± 11.1
Right atrium area, systolic (cm^2^)^a^	16.1 ± 4.5	15.7 ± 4.8	16.5 ± 4.1	15.4 ± 2.5	25.7 ± 2.1
Tricuspid annular plane systolic excursion, TAPSE (mm)	22.1 ± 3.6	21.6 ± 3.1	22.7 ± 4.2	23.3 ± 3.1	18.3 ± 9

Values are presented as means ± SD or *n* (proportion), as appropriate

^a^Evaluated from apical four-chamber view. ^b^Echocardiographic measurements completely or partly available for given *n*

When patients with CHD were excluded, none of the echocardiographic measurements demonstrated statistically significant differences in the comparison of the CS+ and CS− groups.

### Predictors of tricuspid regurgitation

**Fig. 1:**
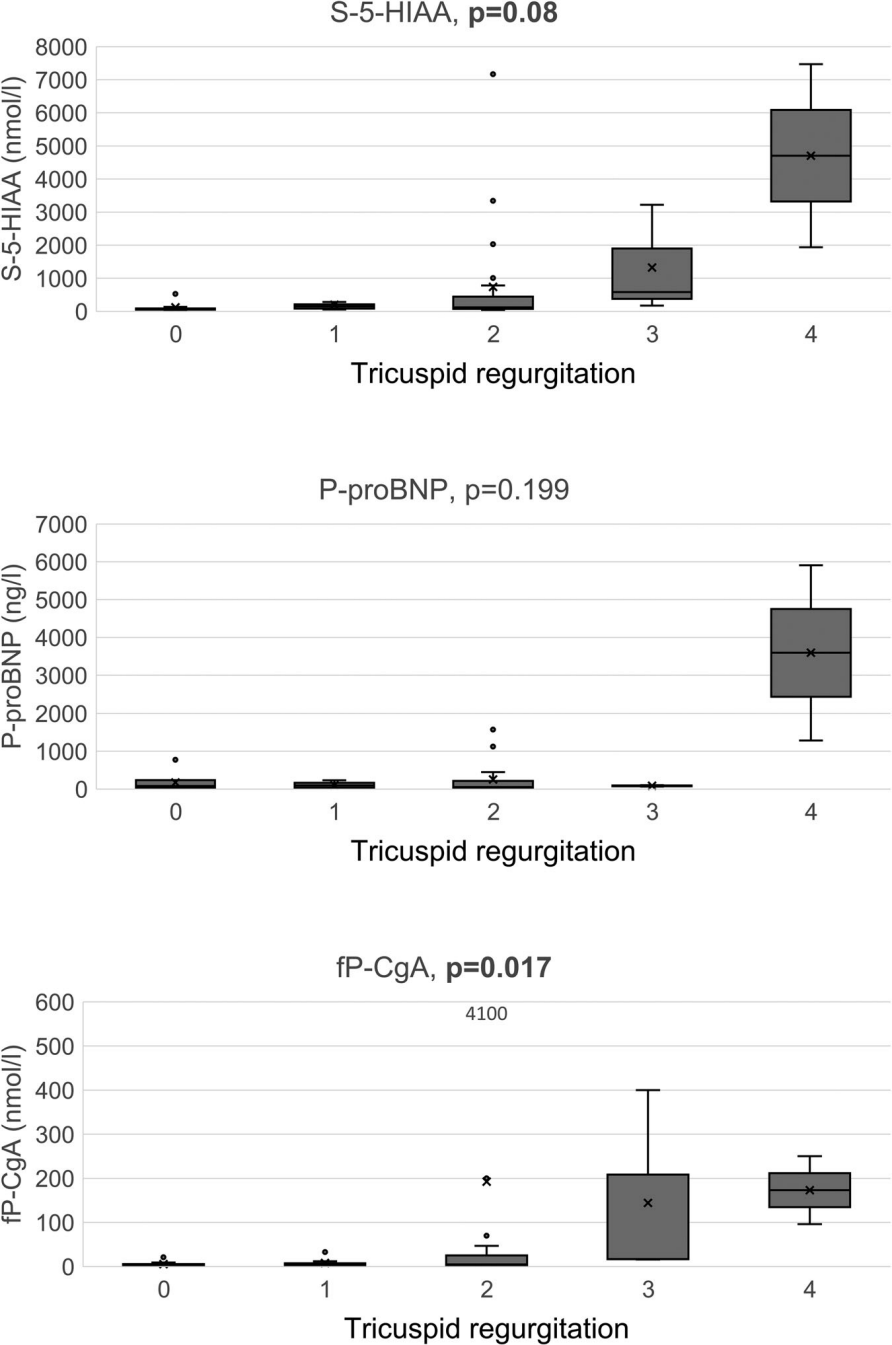
Boxplots of S-5-HIAA, P-proBNP, and fP-CgA by degree of tricuspid valve regurgitation with none/trace/mild/moderate/severe corresponding to 0/1/2/3/4, respectively

The degree of TR was correlated with hepatic tumour load (r = 0.27, *P* = 0.040). There was a statistically significant difference for S-5-HIAA (*P* = 0.017) and fP-CgA (*P* = 0.009) according to the severity of TR (shown in Fig. 1) when all patients were included in the analysis. Both CgA and 5-HIAA increased with more severe TR. However, no such difference was noted for P-proBNP (*P* = 0.199).

To further study the predictors of CHD, we divided the CS− and CS+ subjects into those without (grades 0–1) and with TR (grades 2–4). Subjects with TR of grades 2–4 were characterised by significantly higher hepatic tumour load, fP-CgA, and S-5-HIAA, and lower BMI when compared to TR of grades 0–1 (*P* = 0.049, *P* = 0.033, *P* = 0.030, and *P* = 0.028, respectively). However, the measures of arterial function did not differ between groups stratified by TR grade.

We used a logistic stepwise regression model to study the association of variables with TR. Regurgitation was categorised into two groups: those with no regurgitation and trace regurgitation (grades 0–1) and those with mild or moderate regurgitation (grades 2–3). CHD patients were excluded from the analysis, and thus no study subjects displayed severe (grade 4) mitral regurgitation. The stepwise regression model (R^2^ = 0.255) included sex (*P* = 0.012) and the presence of CS (*P* = 0.041). The model excluded age, BMI, duration of disease, hepatic tumour load, Ki-67 of primary tumour, ejection duration, SEVR, AIx, central systolic pressure, central diastolic pressure, central pulse pressure, central end-systolic pressure, brachial and aortic PWV, RHI, removal of primary tumour, use of somatostatin analogues, PRRT, fP-CgA, and P-proBNP.

## Discussion

We here report, for the first time, simultaneous measurements of echocardiography and arterial and endothelial function in patients with SI-NETs. Further, we describe the prevalence of CS and CHD in our cohort of patients who had received contemporary treatments at a tertiary centre. Our results indicate that metastatic disease and CS are highly prevalent among such SI-NET patients, affecting almost 50% of the patients. However, CHD was found to be less common than previously reported [5, 6, 11, 28]. In the present study, those with grades 2–4 TR were characterised by high liver tumour burden and high levels of S-5-HIAA and fP-CgA. However, we could not identify specific predictors for TR among the detailed vascular function tests performed, nor could such predictors be identified by echocardiography.

CS is characterised by increased concentrations of circulating vasoactive substances, most importantly serotonin, that, besides affecting mood, satiety, and gastrointestinal function, is known to be a key regulator of vascular tone, possessing both vasoconstrictive and vasodilatory properties [14]. Increased serotonin concentrations have been described in cases of arterial hypertension, carotid atherosclerosis, and coronary artery disease [32]. However, its role in vascular pathology in SI-NET—other than right-side valvular disease—is not well known. We utilised robust, validated methodologies to assess arterial (applanation tonometry) and endothelial function (PAT) and the degree of atherosclerosis (CIMT). Surprisingly, we did not observe clear differences in these measures between those with and without CS. The results are interesting and suggest that different pathophysiological mechanisms may be taking place on top of those found in typical cardiovascular disease. However, the findings need to be replicated, and further studies in other cohorts are needed to gain more insights into the mechanisms involved. Of note, subjects with CS had higher central systolic, mean, and systolic BP compared to those not having CS, indicating that CS is characterised by elevated pressure circumstances in the large arteries.

In contrast, the three subjects diagnosed with CHD demonstrated decreased central arterial pressure and an approximately 50% decrease in the augmentation index as compared to those without CHD. Our data suggest that these hemodynamic changes develop after the onset of severe TR in CHD. However, as the number of patients with CHD was small, these findings need to be confirmed.

The CS− and CS+ groups did not differ according to cardiovascular risk factors. Assessed factors included smoking status, LDL cholesterol, previous diagnosis of diabetes mellitus, hypertension and coronary artery disease (data not shown). The carotid IMT was comparable between the groups, suggesting that high serotonin levels or CS are not associated with accelerated vascular atherosclerosis.

In further analysis, we did not detect any differences in vascular function or carotid atherosclerosis between subjects with grades 0–1 or 2–4 TR, suggesting that the risk of CHD development is not related to the vascular function parameters measured in the present study. Therefore, endothelial function tests or systemic vascular resistance measures are not able to discriminate the early asymptomatic phase of CHD, at least in a cross-sectional setting.

Neuroendocrine tumour cells secrete CgA and serotonin, the precursor of 5-HIAA, which thus reflect the disease burden [4] while proBNP is a marker of cardiac failure, mainly secreted by atrial and ventricular myocytes in response to cardiac wall stress [33]. In many studies in patients with neuroendocrine tumours with liver metastases and/or CS, transthoracic echocardiography findings have been correlated with 24-h urinary 5-HIAA levels, but serum N-terminal proBNP has shown more mixed results [20]. Our findings confirm the previous findings that CS and CHD are characterised by increased hepatic tumour burden and concentrations of fP-CgA. Chronic and excessive exposure to circulating serotonin is considered one of the most critical factors contributing to CHD [6, 34]. Since most of the studies have evaluated 24-h urinary 5-HIAA levels [11, 28, 35–39], it must be noted that only a few have reported plasma 5-HIAA concentrations in CHD, and our results validate these findings [40]. In the present study, increases of fP-CgA and S-5-HIAA were statistically significantly associated with the degree of TR, whereas increases of proBNP were not. Our data suggest that follow-up echocardiography is the most sensitive means to identify CHD among patients with SI-NET characterised by significant hepatic tumour burden and increased fP-CgA and 5-HIAA. Although biomarkers are convenient, our study found no single screening marker for early phase of CHD [41]. There is an increasing armamentarium of biomarkers with potential prognostic utility in SI-NET, but further studies are needed to establish their role in CHD [42].

CHD is typically diagnosed 1.5 to 2 years after the diagnosis of SI-NET [11, 19]. Our patient cohort had a rather long disease duration of 76 months. Despite multiple treatment modalities, almost 90% of the patients had at least locally advanced disease and 45% had CS. Regardless of the long disease duration and metastasis, the prevalence of CHD among these SI-NET patients was low at a rate of only 4.6%. A recent systematic review indicated that the incidence of CHD varies widely from 3% to 65% between studies, with older studies tending to report higher estimates of prevalence and incidence. This could reflect more effective options used in the treatment of advanced SI-NET and CS in the more recent studies [20].

The therapeutic options to treat CHD are limited. They consist of tumour debulking surgery, medication to reduce serotonin concentrations (i.e., somatostatin analogues), PRRT, and treatments for right-sided heart failure, including valve surgery. The diagnosis of CHD is associated with a diminished prognosis when compared to SI-NET patients without CHD [20], and the mortality of CHD patients is also high following valve surgery [43]. Early recognition of SI-NET patients with high risk for CHD is thus important to improve their prognosis. In our study, almost 90% of the patients used somatostatin analogues that have efficacy in preventing CHD [44]. Furthermore, 37% of the study subjects had received PRRT, which may be even more effective than somatostatin analogues for the treatment of CS [45]. The use of PRRT was more than double among CS+ , which, together with a large entity of contemporary treatments for SI-NET, may be linked to a lower prevalence of CHD than that reported in historical cohorts.

Our study has some limitations, which include the limited number of patients and the cross-sectional design. Also, the disease duration and treatment modalities may have affected the outcome, as there are reports that SI-NET treatments may decrease 5-HIAA levels, but CHD may still progress [11]. Another problem is that the definition of CS varies between studies and uniform diagnostic criteria are awaited [46].

In summary, we have demonstrated that although CS was highly prevalent among contemporary SI-NET patients treated with current modern therapies at a tertiary referral centre, CHD was rarely encountered. TR in CS patients was not reflected in the measurements of vascular resistance, PWV, or central arterial pressure. Rather, TR was related to hepatic tumour burden and elevated plasma CgA and 5-HIAA concentrations, i.e., to the severity of CS. However, we found no single ideal novel diagnostic marker for CHD, and thus a high degree of clinical awareness still stands as the key to early recognition of CHD. Transthoracic echocardiography remains the first-line imaging modality for the assessment of the severity of tricuspid and pulmonary valve diseases to establish the diagnosis of CHD.
